# Dynamic Forecasting of Zika Epidemics Using Google Trends

**DOI:** 10.1371/journal.pone.0165085

**Published:** 2017-01-06

**Authors:** Yue Teng, Dehua Bi, Guigang Xie, Yuan Jin, Yong Huang, Baihan Lin, Xiaoping An, Dan Feng, Yigang Tong

**Affiliations:** 1 Beijing Institute of Microbiology and Epidemiology, Beijing, China; 2 State Key Laboratory of Pathogen and Biosecurity, Beijing, China; 3 Department of Mechanical and Mechatronics Engineering, University of Waterloo, Waterloo, Ontario, Canada; 4 Beijing Institute of Biotechnology, Beijing, China; 5 Computational Neuroscience Program, Department of Psychology, Physics, and Computer Science and Engineering; Institute for Protein Design, University of Washington, Seattle, United States of America; 6 Division of Standard Operational Management, Institute of Hospital Management, Chinese PLA General Hospital, Beijing, China; Institut Pasteur, FRANCE

## Abstract

We developed a dynamic forecasting model for Zika virus (ZIKV), based on real-time online search data from Google Trends (GTs). It was designed to provide Zika virus disease (ZVD) surveillance and detection for Health Departments, and predictive numbers of infection cases, which would allow them sufficient time to implement interventions. In this study, we found a strong correlation between Zika-related GTs and the cumulative numbers of reported cases (confirmed, suspected and total cases; *p<0*.*001*). Then, we used the correlation data from Zika-related online search in GTs and ZIKV epidemics between 12 February and 20 October 2016 to construct an autoregressive integrated moving average (ARIMA) model (0, 1, 3) for the dynamic estimation of ZIKV outbreaks. The forecasting results indicated that the predicted data by ARIMA model, which used the online search data as the external regressor to enhance the forecasting model and assist the historical epidemic data in improving the quality of the predictions, are quite similar to the actual data during ZIKV epidemic early November 2016. Integer-valued autoregression provides a useful base predictive model for ZVD cases. This is enhanced by the incorporation of GTs data, confirming the prognostic utility of search query based surveillance. This accessible and flexible dynamic forecast model could be used in the monitoring of ZVD to provide advanced warning of future ZIKV outbreaks.

## Introduction

Zika virus (ZIKV) is transmitted to people primarily by mosquitoes [1]. Prior to 2015, outbreaks had occurred in Africa, Southeast Asia, and the Pacific Islands [2, 3, 4]. In May 2015, the presence of Zika virus disease (ZVD) was confirmed in Brazil. ZIKV has subsequently reportedly been spreading throughout the Americas, with epidemics occurring in many countries [5, 6]. The World Health Organization declared ZIKV, and its suspected link to birth defects, an international public health emergency in February 2016 [7, 8]. Traditional, healthcare-based and government-implemented, ZVD monitoring is resource intensive and slow. Early surveillance of infectious disease prevalence, when followed by an urgent response, can reduce the effects of disease outbreaks [9]. Surveillance of online behavior, such as queries in search engines, is a potential web-based disease detection system that can improve monitoring [10]. Google Trends has been shown to have the potential to go beyond early detection and forecast future influenza and Dengue outbreaks [11–14]. Several studies have used autoregressive integrated moving average (ARIMA) models for the forecasting of influenza prevalence from Google Flu Trends [15, 16]. The real-time nature of GTs surveillance and the demonstrated strong correlation of GTs with infectious disease mean GTs offers a potential tool for timely epidemic detection and prevention [17]. However, the forecasting capabilities of GTs for ZIKV outbreaks remain unknown. In this study, we examined the ZIKV-related GTs temporally correlated with ZVD epidemics, and developed an improved dynamic forecasting method for ZVD activity in the worldwide using an ARIMA model to predict future patterns of ZIKV transmission.

## Materials and Methods

### Data collection and statistical analysis

Google Trends, an online tracking system of Internet hit-search volumes (Google Inc.), was used to explore web behavior related to the ZIKV outbreaks. GTs data for ZIKV in worldwide was mined of the key word “Zika” from 12 February 2016 to 9 November 2016 (Yearly EPI Week 6 to 45) to cover the 2016 period of the ZIKV epidemic, and was downloaded directly from https://www.google.com/trends/explore?date=all&q=zika on 9 November 2016. Although Google Trends normalizes the search data with the day having the most searches set equal to 100, we obtained and analyzed the relative search volumes for each day based on the data (100) in 12 February 2016 (Yearly EPI Week 6; data shown in S1 Table). The number of ZIKV confirmed, suspected and total cases in the worldwide were retrieved from the PAHO (Pan American Health Organization), available at http://www.paho.org/hq/ and WHO (World Health Organization), available at http://www.who.int/emergencies/zika-virus/situation-report/en/ (last accessed on 9 November 2016, S2 Table). To detect the cumulative GTs volumes relative to reported ZIKV cases, we used the Pearson Product-Moment Correlation to assess linear correlation. All calculations were performed in Python 2.7 with the Scipy library.

### Linear regression model

As a baseline model for comparison, the data is fitted with a linear regression model to establish the relationship of the cases to the GTs data. The linear model is constructed with R version 2.14 (http://www.r-project.org/) and the parameters are obtained automatically. Prediction results are plotted together with the proposed ARIMA model to show the comparison outcome.

### Reconstructed ARIMA model

For the time series analysis, we fitted an autoregressive integrated moving average (ARIMA) (0, 1, 3) model by using R version 2.14 (http://www.r-project.org/). The autoregressive integrated moving average (ARIMA) forecasting model in this study was developed from the training and testing sets that were extracted from the data sets. We used the data set including the 9 months from 12 February to 9 November 2016 (Yearly EPI Week 6 to 45) in the analysis. The data from 12 February to 25 August 2016 (Yearly EPI Week 6 to 34) were used for training the forecasting model, and the validation was performed on the 8 weeks’ data from 1 September to 20 October 2016 (Yearly EPI Week 35 to 42). We applied the Box-Jenkins approach to ARIMA (p, d, q) modeling of time series. This model-building process is designed to take advantage of associations in the sequentially lagged relationship that usually exists in periodically collected data [18, 19]. The parameter for the model includes p, the order of autoregression; d, the degree of difference and q, the order of moving average. Several models were initially considered in our study (S3 Table). The residual analysis and the Akaike Information Criterion (AIC) was used to compare the goodness-of-fit among the ARIMA models. The Ljung-Box test was used to measure the ACF of the residuals (S3 Table). Using this model selection procedure, we selected one non-seasonal difference term for stationary (d) and three lags of moving average terms (q), resulting in a model of ARIMA (0, 1, 3). To predict the future values, the developed ARIMA model was fitted to the entire data from 12 February to 20 October 2016 (Yearly EPI Week 6 to 42) and used to forecast over a time span of 3 weeks, covering 27 October and 9 November 2016 (Yearly EPI Week 43 and 45).

## Results

### Correlations between data on ZIKV outbreaks and ZIKV-related GTs

**Fig 1:**
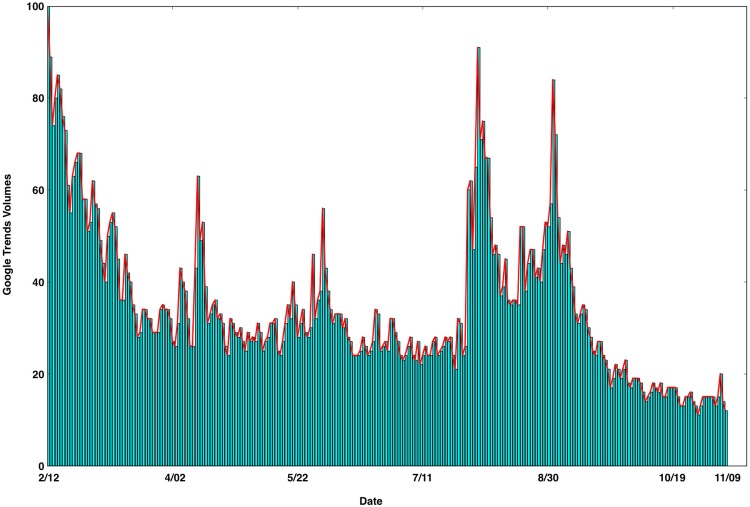
Time series plot of the daily GTs volumes from 12 February to 9 November 2016 (Yearly EPI Week 6 to 45).

**Fig 2:**
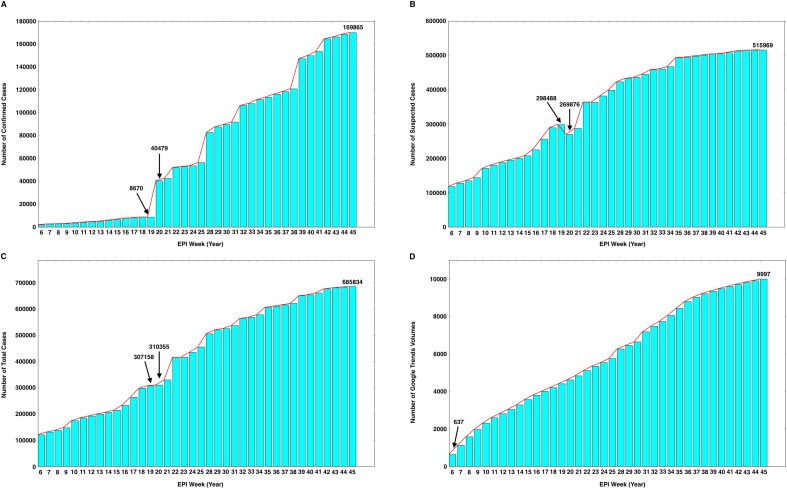
Time series plots of the number of reported ZIKV confirmed cases (A), suspected cases (B), total cases (C) and the accumulated Google search volumes (D) during the ZVD epidemic from 12 February to 9 November 2016 (Yearly EPI Week 6 to 45).

Our analyses used the data from 12 February to 9 November 2016 (Yearly EPI Week 6 to 45), covering 9 months of the GTs data (Fig 1 and S1 Table) and the reported ZIKV epidemic data (confirmed, suspected and total cases, S2 Table). Alongside the "stepped” increases in the reported ZIKV cases (confirmed, suspected and total cases) in this epidemic, there were dramatically increased numbers of Zika-related online searches in Google (Fig 2). From 12 May to 19 May 2016 (Yearly EPI Week 19 to 20), the ZIKA epidemics entered a period of rapid growth with a large cumulative number of confirmed cases (increasing from 8,670 to 40,479; Fig 2A). Meanwhile, the cumulative number of suspected cases fell from 298,488 to 269,876 (Fig 2B), because the suspected cases were diagnosed during this period. Then, the number of total reported cases increased slightly from 307,158 to 310,355 (Fig 2C). Until 20 October 2016 (Yearly EPI Week 42), a total of 164,352 confirmed cases and 512,345 suspected cases were reported in the S2 Table. The GTs data showed that the volume of dynamic Zika-related online searches continuously grew from February to November 2016 (Fig 2D). We performed Pearson Product-Moment Correlation analyses to examine temporal correlations between the accumulative volumes of Zika-related search queries and the cumulative numbers of reported cases. The result indicated that the data on Zika-related GTs had statistically significant and positive correlations with the cumulative numbers of confirmed cases (R = 0.968, *p<0*.*001*), suspected cases (R = 0.980, *p<0*.*001*) and total cases (R = 0.988, *p<0*.*001*) of ZIKV.

### Statistical machine learning and reconstructed ARIMA model

**Fig 3:**
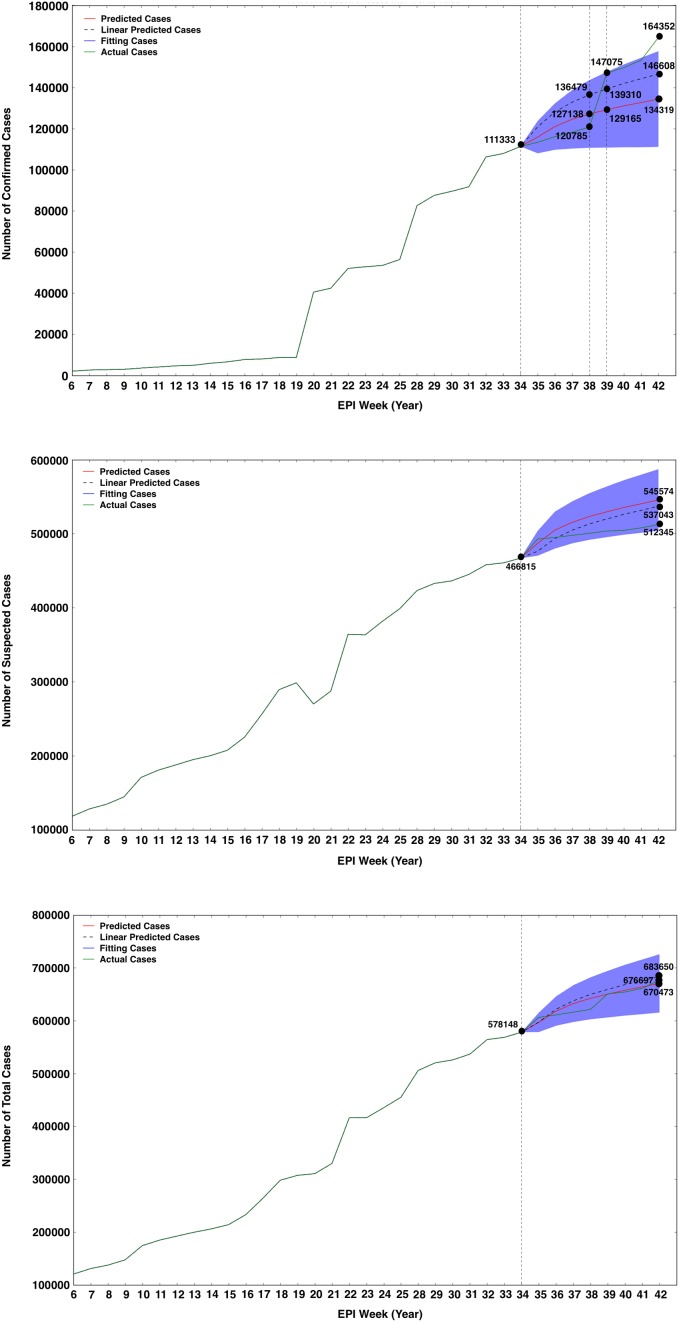
Numbers of reported confirmed cases (A), suspected cases (B) and total cases (C) in the testing set compared with the simulation data by the advanced ARIMA (0, 1, 3) model for training set using the data of Google Trends as the external regressor. The red, blue and green solid lines represent the predicted, training and actual number of cases, respectively. The black dash lines represent the prediction of the linear baseline model. The blue region represents the 95% confidence interval predicted by the ARIMA (0, 1, 3) model.

On the basis of the revealed correlation between the data of GTs and the number of reported cases, we split the data into training (75%, from 12 February to 25 August 2016) and testing (25%, from 26 August to 20 October 2016) sets. We analyzed the training set to observe whether the simulation data is similar to the reported data in testing set using the advanced autoregressive integrated moving average (ARIMA (0, 1, 3)) model. In this reconstructed model, we used the collected online search data as the external regressor in a prediction model to assist the historical ZVD epidemic data in improving the quality of prediction. Using this model with the training data of GTs as a predictor, we found that the cumulative number of reported confirmed case (164,352) in 20 October 2016 (Yearly EPI Week 42) in the testing set was above the 95% confidence interval of the simulated data (111,119 to 157,663; Fig 3A). However, Fig 3B and 3C showed that the number of suspected cases (512,345) and the number of the total cases (676,697) in 20 October 2016 (Yearly EPI Week 42) in the testing set fell within the 95% confidence interval of the simulated data (504,034–587,115 for suspected cases and 615,347–725,599 for total cases, respectively). We also used a simple baseline model to do a sanity test as a reference, which could reproduce features in the data and predicted the number of confirmed, suspected and total cases in 20 October 2016 (Yearly EPI Week 42; 146,608 for confirmed cases, 537,043 for suspected cases and 683,650 for total cases in Fig 3A, 3B and 3C). However, the complicated ARIMA model showed a better performance for the prediction with a lower value of AIC than the simple linear predictor (S3 Table). These observations implied that the Google Trends information would improve the prediction of the size of ZIKV outbreaks.

### Forecasting the ZIKV outbreaks in worldwide during early november 2016

**Fig 4:**
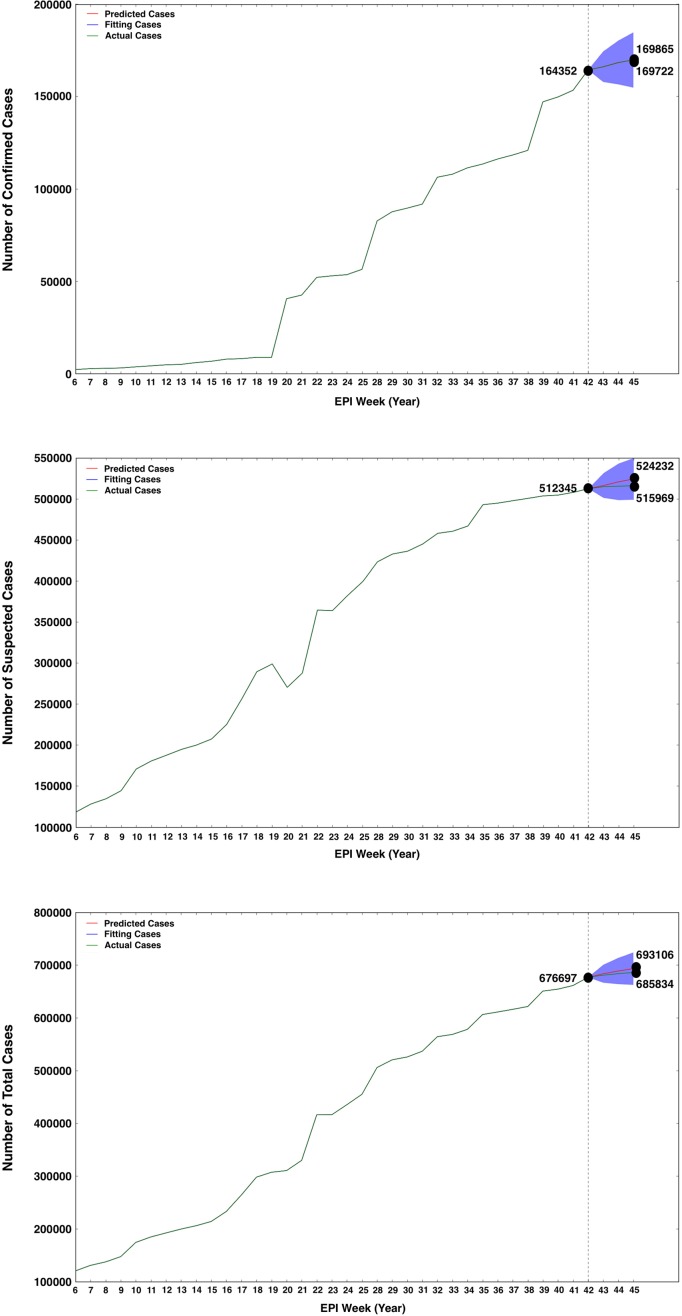
Forecasts of the number of ZIKV confirmed cases (A), suspected cases (B) and total cases (C) in worldwide between 27 October and 9 November 2016 (Yearly EPI Week 43 and 45) by the advanced ARIMA (0, 1, 3) model, which was improved by aggregating historical logs with the most-current 3 weeks’ data of Zika-related Google Trends as a estimating predictor to estimate ZVD cases. The red, blue and green solid lines represent the predicted, training and actual number of cases. The blue region represents the 95% confidence interval predicted by the ARIMA (0, 1, 3) model.

Based on the data on Zika-related online searches and ZIKV epidemics in GTs between 12 February and 20 October 2016, we used the reconstructed ARIMA (0, 1, 3) model to forecast ZVD outbreaks during the current 3 weeks. In this model, we used the online search data as the external regressor to enhance the forecasting model and assist the historical ZVD epidemic data in improving the quality of the predictions while responding to disease outbreaks. We forecasted the cumulative number of ZIKV cases as a simulation of the continuation of the time series. This model with GTs data as the predictor estimated that the forecast cumulative number of confirmed cases would increase to reach 169,722.4 (95% CI: 154,746.8–184,698.0) by 9 November (Yearly EPI Week 45), which was closest to the actual reported infection cases (169,865; Fig 4A). The predicted number of suspected cases would grow up to 524,231.6 (95% CI: 498,863.8–549,599.4) on 9 November 2016 (Yearly EPI Week 45; Fig 4B), which was slightly more than the reported suspected cases (515,969). Finally, the actual number of total reported cases was 685,834 on 9 November 2016 (Yearly EPI Week 45) and the predicted number of total cases was 693,106 (95% CI: 662,396.1–723,815.9) (Fig 4C), which was above the reported cases (S4 Table). These forecasts of ZVD outbreaks suggested that ZIKV disease transmission in the worldwide remains intense during November 2016.

## Conclusions

ZVD outbreaks are now a common and growing problem worldwide [20, 21 22]. Delays in traditional surveillance systems limit the ability of public health agencies to respond efficiently to ZIKV epidemics [23]. Because data on GTs are collected and processed in near real-time, online search information produces monitoring data much faster than traditional systems [24, 25]. We first performed correlation analyses to investigate the temporal correlations between data on ZIKV-related GTs and reported cases of ZVD. The result showed that the Zika-related GTs had a strong correlation with confirmed, suspected and total cases of ZIKV. Based on the correlation data, the advanced model ARIMA (0, 1, 3) was improved by aggregating historical logs and estimated data of online search queries associated with Zika as a predictor to forecast ZVD cases. And we found that the complicated ARIMA model showed a good performance for the prediction with the lower value of AIC than the simple linear predictor (Fig 3). The results also indicated that the predicted data by ARIMA model are quite similar to the actual data during ZIKV epidemic early November 2016 (Fig 4). The results in this study showed that the novel surveillance tool of GTs can also provide dynamic timely information to public health agencies and provide near real-time indicators of the spread of infectious disease [26]. However, to be effective for monitoring disease activity on local geographic areas, it must be considered within the local context of ZIKV transmissibility.

## Supporting Information

S1 Table Daily Google Trends Volumes from 12 February to 9 November, 2016 (Yearly EPI Week 6 to 45). The search region is set to ‘Worldwide’ and the key wrod is the ‘Zika’.
(XLSX)

S2 Table Number of reported confirmed cases, reported suspected cases, reported total cases and cumulative Google Trends volumes about ZIKV in the worldwide during the ZVD epidemic of 12 February to 9 November 2016 (Yearly EPI Week 6 to 45; 39 weeks). (XLSX)

S3 Table Comparison of tested models. (XLSX)

S4 Table Predictions of the numbers of confirmed, suspected and total cases from 27 October to 9 November 2016 (Yearly EPI Week 6 to 45) by the advanced ARIMA (0, 1, 3) model, which was improved by aggregating historical logs with data of Zika-related Google Trends as an estimating predictor to forecast ZVD cases. (XLSX)
